# Supplementary material for: WHAMM initiates autolysosome tubulation by promoting actin polymerization on autolysosomes
Source: Nat Commun. 2019 Aug 16;10:3699. doi: 10.1038/s41467-019-11694-9 (PMC6697732; doi:10.1038/s41467-019-11694-9)
Supplement: Supplementary file 1 — Supplementary Information [file 41467_2019_11694_MOESM1_ESM.pdf]

Supplementary Information

WHAMM initiates autolysosome tubulation by promoting actin polymerization on autolysosomes

Dai et al.

Supplementary Figures

a

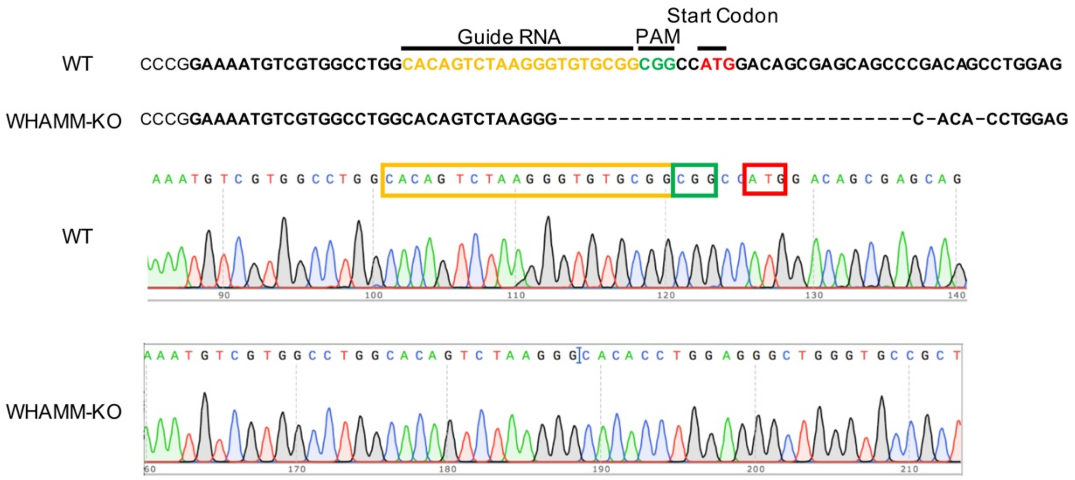

b

	Target sequence with PAM (N = mismatch)	Genomic Site	Primers	Modifications
On-target	CACAGTCTAAGGGTGTGCGGCGG	Chr 1: 143,402,003-143,402,023	CCTGCCTCCCCGAAAAGAC<br>GTCTTTTCGGGGAGGCAGG	20/20
Off-target 1	CCCAGTGCAAGGCTGTGCGGTGG	Chr 9: 43,113,181-43,113,206	CAAGTACCTGGCAGACGGAG<br>CAGGTTACCACCCAGAGCAG	0/20
Off-target 2	CACAGTCCATGTGTGTGCGATGG	Chr 1: 198,775,384-198,775,409	CAGGCCCTAGAACTCACCCA<br>CTTTGCCCTCTTCTGAGTGGTA	0/20
Off-target 3	CACAGCCCAGGGGTGTGCTGGGG	Chr 1: 198,259,842-198,259,867	TCCTATCCAAGAACACGCTCC<br>CAAGGAGGAAGGAAGCTTGG	0/20
Off-target 4	TACAGCCTCAGGGTGAGCGGTGG	Chr 10: 39,039,927-39,039,952	GGTAAGCTGTCCAGGTCACAA<br>GCTTGAAAGTCTGAGCCCAAG	0/20
Off-target 5	CACAGTCTACTGGTGTCGGGAGG	Chr 14: 20,149,590-20,149,615	TGCCTCACTGACATGTACCTC<br>ATAGTGGCAAAGAGCACGGT	0/20
Off-target 6	CATAGCCTAAGGGTGAGCTGTGG	Chr 6: 92,661,888-92,661,913	GCCAGGCTTTTATCGCAGCTA<br>GAAGTGGGAACACACGGGAG	0/20
Off-target 7	CACAGTCTGGGGGTGGGAGGGGG	Chr 7: 119,663,414-119,663,439	AGCACTGTGGTGAGCCTAGT<br>AAGCAGTGAGCGGCTGTTAT	0/20

Supplementary Figure 1 Generation of the WHAMM-KO NRK cell line by CRISPR-Cas9 mediated gene knockout.

(a) The guide RNA and PAM sequence are highlighted. DNA sequencing results are shown from a single colony, with the same modification successfully introduced into both alleles. (b) Potential off-target sites were analyzed using the same PCR based method as on-target site. 20 colonies were

selected and sequenced to verify modification.

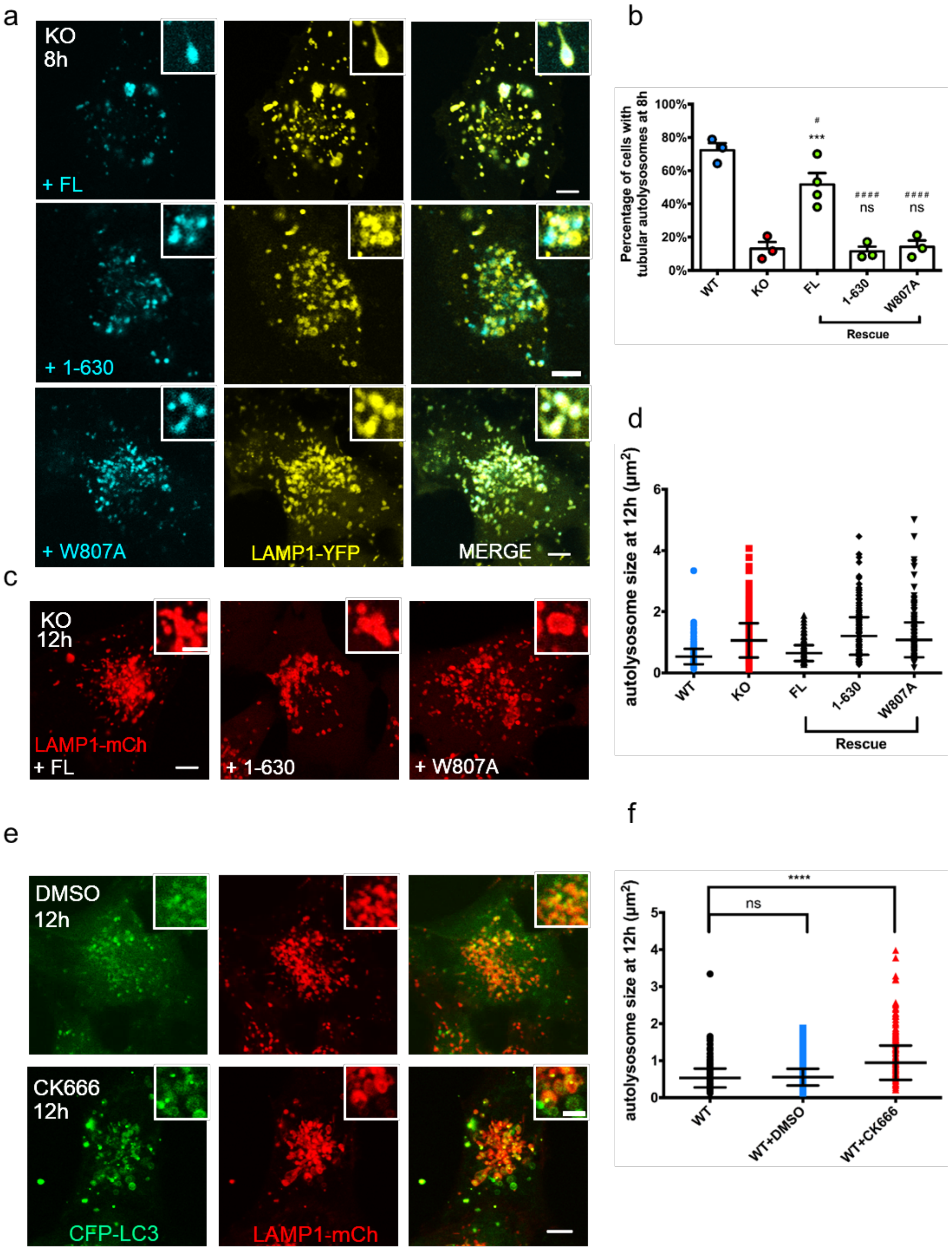

Supplementary Figure 2 WHAMM's function as an NPF is required in ALR.

(a) Relative staining of WHAMM (FL and mutants) with autolysosomes from Fig 3b. WHAMM and its mutants were pseudo-colored to cyan. (b) Cells in (a) were quantified for tubular

autolysosomes. A total of 103 (WT), 120 (KO) 81 (1-630) and 88 (W807A) cells were analyzed in n=3 independent experiments. 125 (FL) cells was analyzed in n=4 independent experiments. Error bars indicate SEM. One-way ANOVA followed with Holm-Sidak's multiple comparisons test. Compared with KO: ***, p<0.001; ns, not significant. Compared with WT: ####, p<0.0001, #, p<0.05. (c) Cells in (a) were further starved to 12 hours and observed using confocal microscopy. Enlarged autolysosomes can be seen in mutant rescue cells (scale bar, main micrograph 5 μm; upper panel 2 μm). (d) The size of autolysosomes from cells in (c) was measured. n=799 (WT), 824 (KO), 310 (FL), 618 (1-630) and 514 (W807A) autolysosomes from three independent experiments. Error bars indicate SD. (e) Cells treated with CK666 were further starved to 12 hours. Accumulation of large autolysosome can be observed in drug-treated cells (scale bar, main micrograph 5 μm; upper panel 2 μm). (f) The size of autolysosomes from cells in (e) was measured. n=799 (WT), 750 (DMSO) and 1074 (CK666) autolysosomes. Error bars indicate SD. One-way ANOVA followed with Holm-Sidak's multiple comparisons test. ****, p<0.0001; ns, not significant. Source data are provided as a Source Data file.

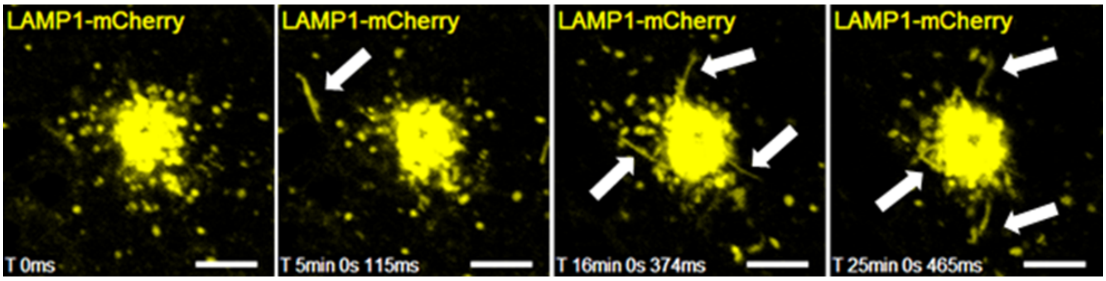

Supplementary Figure 3 Recovery of tubulation events after CK666 wash-out.

Scale bar, 5 µm. LAMP1-mCherry was pseudo-colored to yellow.

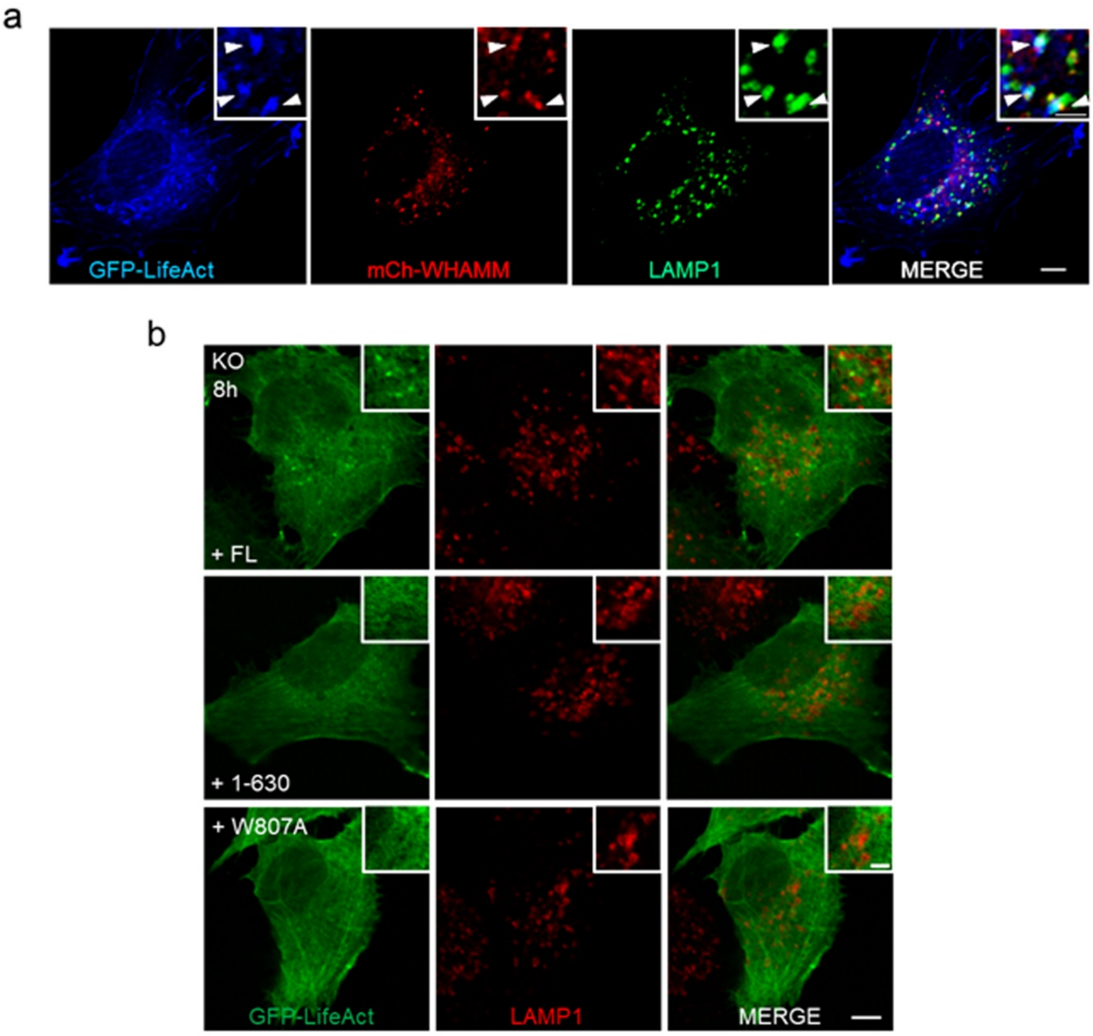

Supplementary Figure 4 WHAMM's NPF activity is required for F-actin formation on autolysosomes.

(a) WT NRK cells stably expressing mCherry-WHAMM were transfected with GFP-LifeAct. 18 hours post-transfection, cells were starved for 4 hours, then fixed and stained with antibody against LAMP1. Arrowheads indicate co-localized WHAMM and LifeAct on an autolysosome (scale bar, main figure 5 μm; upper right panel 2 μm). (b) WHAMM-KO cells were transfected with GFP-LifeAct and constructs containing WHAMM-FL or different NPF-defective mutants. 18 hours post-transfection, cells were starved for 8 hours and fixed and stained with antibody against LAMP1 (scale bar, main figure 5 μm; upper right panel 2 μm).

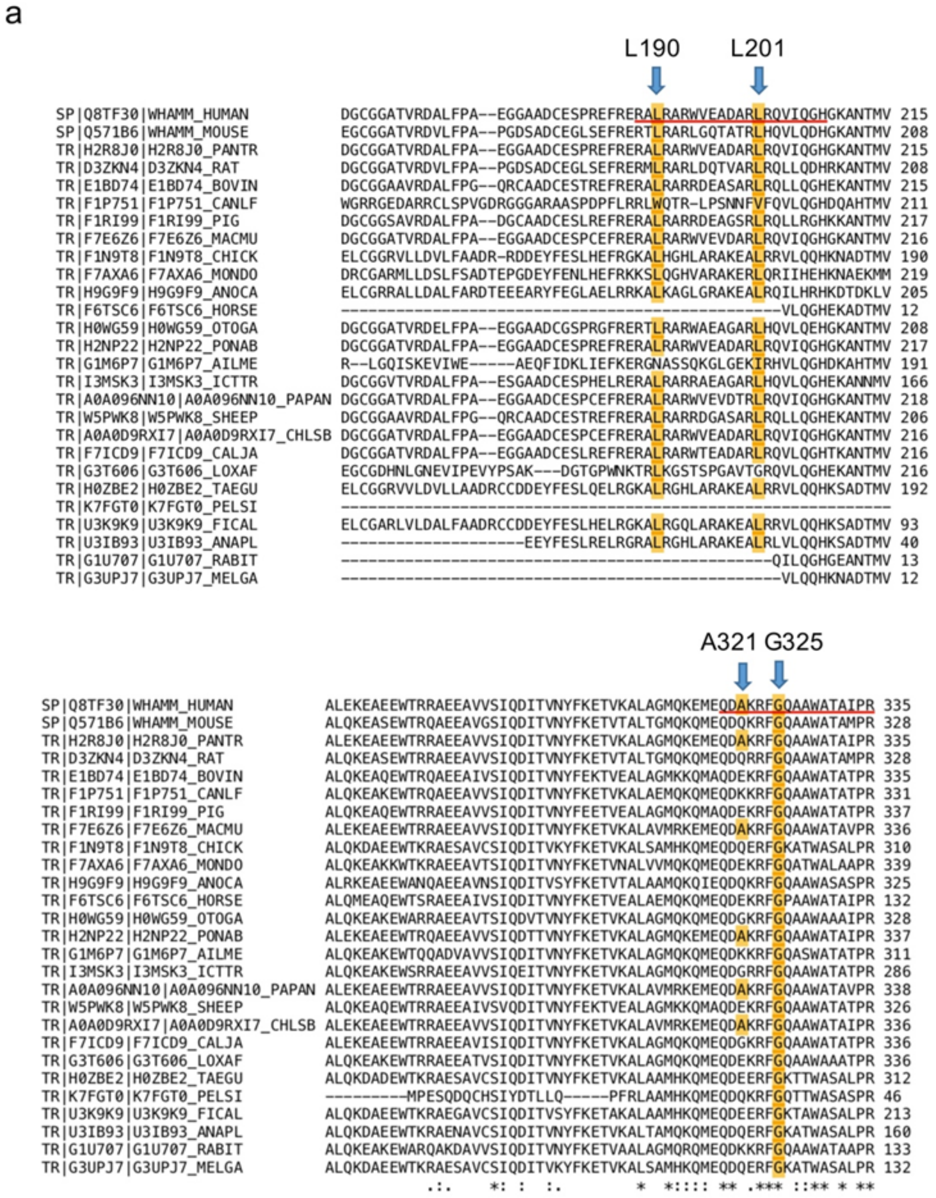

Supplementary Figure 5 WHAMM binds to PI(4,5)P_2 through two conserved amphipathic helices.

(a) Sequence alignment of the regions contain the two amphipathic helices. The helices are underlined in red. Sites changed by mutagenesis in this work are indicated with an arrow and highlighted in yellow.

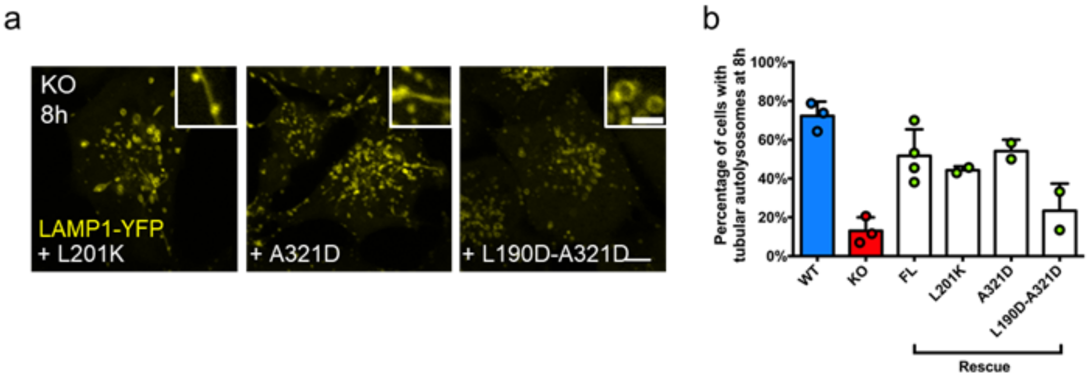

Supplementary Figure 6 WHAMM's interaction with PI(4,5)P_2 is required for ALR.

(a) FL WHAMM with single mutations (L201K, A321D) or another double mutation (L190D-A321D) were transfected into WHAMM-KO cells stably expressing LAMP1-YFP. 18 hours post-transfection, cells were starved for 8 hours and observed using confocal microscopy (scale bar, main micrograph 5 μm, upper panel 2 μm). (b) Cells in (a) were quantified for tubular autolysosomes. A total of 103 (WT), 120 (KO) and 105 (FL) cells were examined in n=3 independent experiments. 84 (L201K), 78 (A321D) and 103 (L190D-A321D) cells were examined in n=2 independent experiments. Source data are provided as a Source Data file.

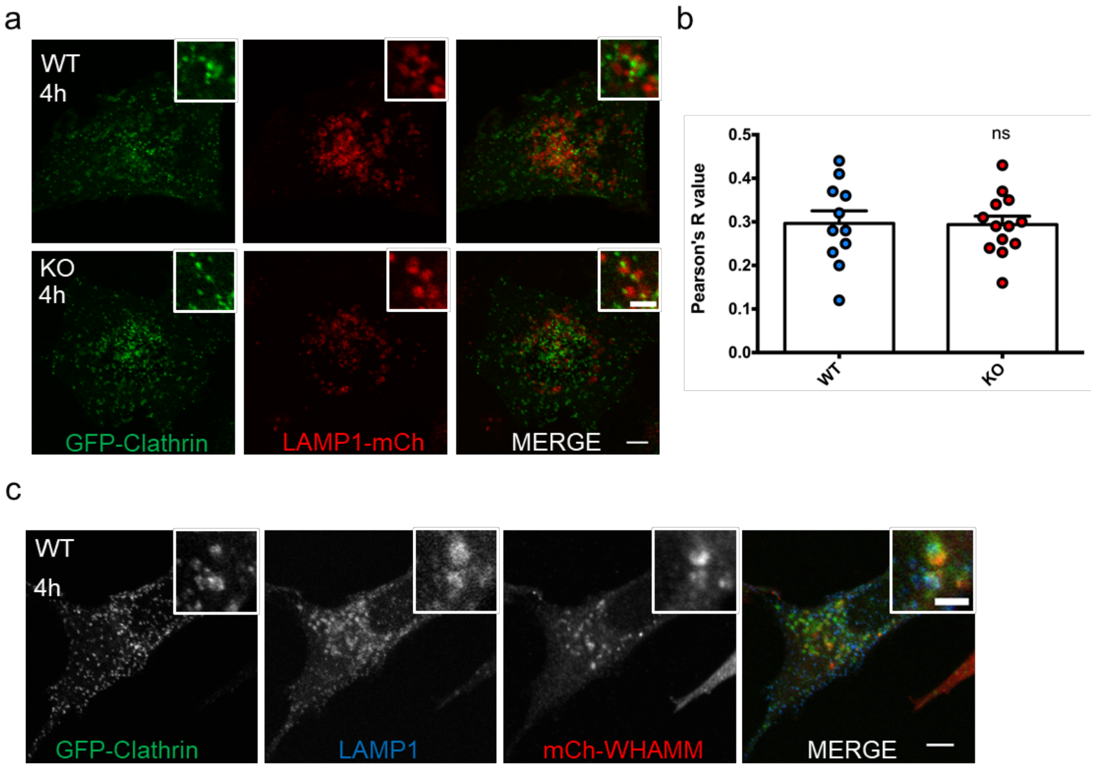

Supplementary Figure 7 WHAMM may collaborate with Clathrin on autolysosomes.

(a) Both WT and WHAMM-KO cells were co-transfected with GFP-Clathrin light chain A and LAMP1-mCherry. 18 hours post-transfection, cells were starved for 4 hours and observed using confocal microscopy (scale bar, main micrograph 5 µm; upper right panel 2 µm). (b) Pearson's co-localization coefficient was calculated for cells from (a) using ImageJ. n=11 (WT) and 13 (KO) cells from two independent experiments. Error bars indicate SEM. Two-tailed t test; ns, not significant. (c) GFP-Clathrin light chain A was transfected into WT NRK cells stably expressing mCherry-WHAMM. Cells were starved for 4 hours, then fixed and stained with LAMP1 antibody (scale bar, main micrograph 5 µm; upper right panel 2 µm). Source data are provided as a Source Data file.

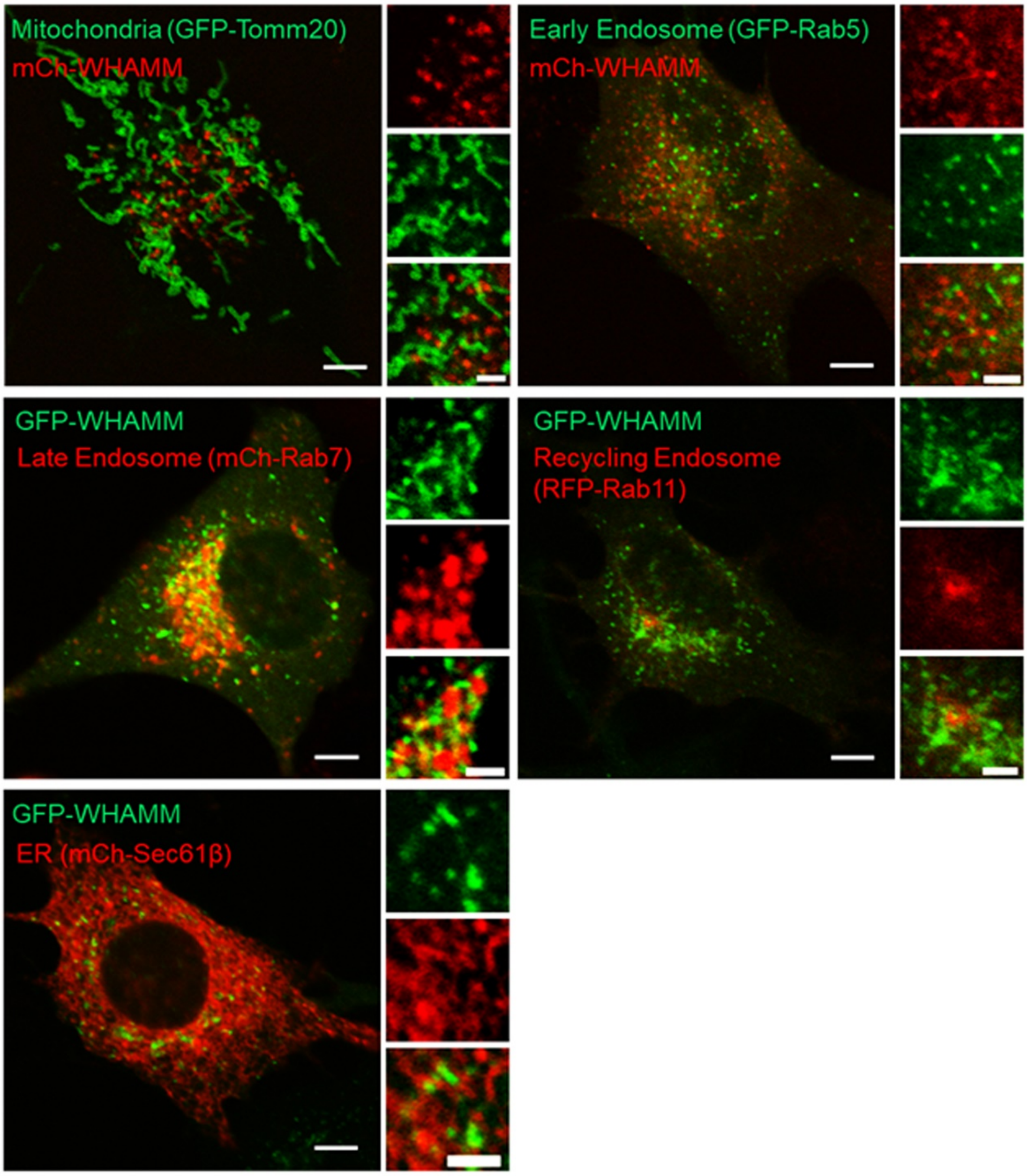

Supplementary Figure 8 Subcellular localization of WHAMM before autophagy induction in NRK cells.

(a) Constructs expressing markers for each designated cellular compartment were transfected into cells stably expressing GFP-WHAMM or mCherry-WHAMM. 18 hours post-transfection, cells were observed under a confocal microscope (scale bar, main figure 5 μm; right panel 2 μm).

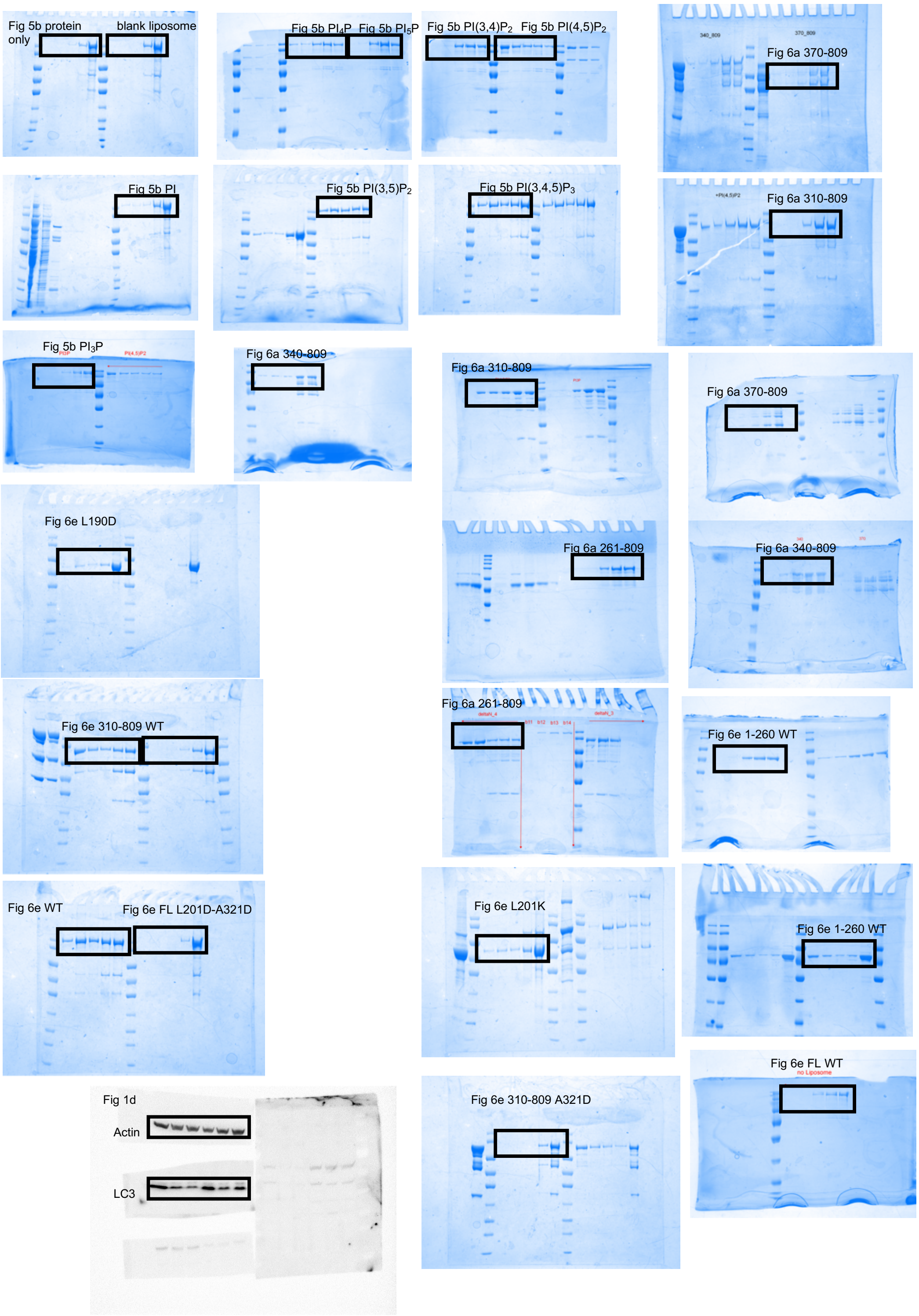

Supplementary Figure 9 Uncropped gels and blots.

Supplementary Tables

Supplementary Table 1 List of primers used in this paper.

Primer	Sequence (5'-3')
Arp3-F	GGAATTCTATGGCGGGACGGCTGCCGG
Arp3-R	ggGGTACCTTACGACATGACTCCAAAC
Cortactin-F	GGAATTCTATGTGGAAAGCTTCAGCAGG
Cortactin-R	ggGGTACCCTACTGCCGCAGCTCCACATAG
F-Mlu1-MBP	CGACGCGTatgaaaactgaagaaggt
F-EcoR1	GGAATTCTATGGAGGACGAGCAG
260-R	ATAAGAATGCGGCCGCTTACTAAATATCAAGCTTACA
630-R	ACG CGT CGA CCT AGG AAT GTG TTT GAT
809-R-Not1	ATAAGAAT<u>GCGGCCGC</u>TTActaACCATCCCACTGGCC
809-R-Kpn1	ggGGTACCTTActaACCATCCCACTGGCC
261-F	CGGGATCCTTGAAGTCTTTGGATG
310-F	CGACGCGTttagcaggaatgcag
340-F	CGACGCGTcagctaatgctagctcgag
370-F	CGACGCGTgaagatcttccagaac
L201D-F	GGGTCGAGGCGGACGCGCGGGACCGCCAGGTTATTC
L201D-R	GTCCCGCGCGTCCGCCTCGACCCACCGCGCGCG
L201K-F	GGGTCGAGGCGGACGCGCGGAAGCGCCAGGTTATTC
L201K-R	CTTCCGCGCGTCCGCCTCGACCCACCGCGCGCG
L190D-F	GCGAGTTCCGGGAGCGGGCCGACCGCGCGCGGTGG
L190D-R	GTCGGCCCGCTCCCGGAACTCGCGCGGGCT
A321D-F	GCAGAAAGAAATGGAACAGGATGATAGAGATTTGGT
A321D-R	ATCATCCTGTTCCATTTCTTTCTGCATTCCTGC
W807A-F	GATGAGCAGGACCCTGGCCAGTGGGAGCGTTAAGGTAC
W807A-R	CGCCTGGCCAGGGTCCTGCTCATCACTGTCCTC
